# Comparison of methods for quantitative biomolecular interaction analysis

**DOI:** 10.1007/s00216-021-03623-x

**Published:** 2021-09-10

**Authors:** Monika Conrad, Peter Fechner, Günther Proll, Günter Gauglitz

**Affiliations:** Institute of Physical and Theoretical Chemistry (IPTC), Eberhard Karls Universität Tübingen, Auf der Morgenstelle 18, 72076 Tübingen, Germany

**Keywords:** Reflectometric interference spectroscopy, Biomolecular interaction analysis, Binding kinetics, Association rate constant, Pseudo-first-order kinetics

## Abstract

In order to perform good kinetic experiments, not only the experimental conditions have to be optimized, but the evaluation procedure as well. The focus of this work is the in-depth comparison of different approaches and algorithms to determine kinetic rate constants for biomolecular interaction analysis (BIA). The different algorithms are applied not only to flawless simulated data, but also to real-world measurements. We compare five mathematical approaches for the evaluation of binding curves following pseudo-first-order kinetics with different noise levels. In addition, reflectometric interference spectroscopy (RIfS) measurements of two antibodies are evaluated to determine their binding kinetics. The advantages and disadvantages of the individual approach will be investigated and discussed in detail. In summary, we will raise awareness on how to evaluate and judge results from BIA by using different approaches rather than having to rely on “black box” closed (commercial) software packages.

**Supplementary Information:**

The online version contains supplementary material available at 10.1007/s00216-021-03623-x.

## Introduction

The analysis of biomolecular interaction is a fascinating field of research. Especially important is the analysis of binding kinetics, which allows the determination not only of the thermodynamic affinity constant, but also of the kinetic association and dissociation rate constants. The knowledge of these constants leads to a deeper understanding of how biological systems function at the molecular level, which can be very useful for pharmaceutical research and rational design of therapeutics [1].

Biomolecular interaction analysis (BIA) aims to quantify interaction patterns in order to describe events between biomolecules, e.g. antibody and its antigen. The analysis of binding events is error-prone because of the user influence on experimental design, on the used analytical method, on the quality of measurement, and on data evaluation. As one-to-one interaction is the simplest model available, it is often applied. However, the experimental setup must be designed carefully to make sure the chosen mathematical model is applicable. Since it is a difficult task to achieve good experimental design, there are reviews pointing out that the quality of published biosensor work is often poor [2, 3]. But there are also publications demonstrating how good kinetic analysis should be performed [4–6]. For example, a study with different Biacore users showed that it is possible to obtain reproducible kinetic constants with proper instructions [7].

The principle of BIA is to detect time-resolved specific interaction of an analyte in continuous flow with an immobilized ligand [8]. Many papers determine binding constants or rates of interactions in immunology [9, 10], drug screening [11, 12], or even proteomics research [13, 14]. Especially suitable for BIA are label-free techniques as they avoid disturbances from conjugated markers or complex handling of radioactive material. Common techniques to measure label-free binding are isothermal titration calorimetry (ITC) [15] or surface plasmon resonance (SPR) [16]. SPR belongs to the direct optical detection methods [17, 18] alongside integrated optical grating coupler [19] or reflectometric interference spectroscopy (RIfS) [20]. They allow time-resolved measurements yielding thermodynamic and kinetic information. However, as mentioned in a recent review [21] matrix, both instrumentation and flow influence measurements.

Commercial software for data evaluation is available, e.g. from Biacore [22], TraceDrawer [23], or Scrubber [24]. Besides, there is open-source software available like Anabel [25] or EvilFit [26]. A problem when using available software is that it is irrelevant whether the user understands what exactly the software does, which might lead to wrong application and consequently to false constants. The most important question the user should ask himself is whether the assumed model is correct. This requires an understanding of biomolecular processes in the homogeneous phase, of transport processes to or from the surface, and of the kinetic processes at the biosensor [27]. RIfS belongs to the heterogeneous immunoassays; thus, the immune reaction takes place on a solid phase with an immobilized component and ideally only effects at the surface are monitored.

Kinetic analysis of binding curves is a long-established procedure. Often the complex binding process is reduced to the reaction between the immobilized ligand and the analyte in solution which represents one-to-one kinetics. This one-to-one interaction can be described by pseudo-first-order kinetics, if the analyte flows over the surface resulting in its concentration remaining constant. Assuming one-to-one interaction, the rate and affinity constants can be easily calculated, if the association and dissociation curves are measured for various analyte concentrations. Experimental conditions should be adapted to avoid deviations from the pseudo-first-order kinetic model as described in [5]. Mass transport limitation, for example, can be reduced by using fast flow rates and by reducing the immobilization level of the recognition element. It can be verified by varying the flow rate [28].

For the calculation of the rate constants, different mathematical approaches can be used: linear transformation of the primary data by use of the derivative [9] or by use of the integral of the binding curve, or the integrated rate equation, which gives an exponential function [29]. There are several factors that can prevent the binding curve to follow pseudo-first-order kinetics: mass transfer [30], rebinding of analyte [31], bivalency or even other orders of analyte, two-state reaction [10], parallel reactions, or competing reactions [32]. Some of these effects can be ruled out by a careful experimental design, but it is also possible to apply more complex models, if numerical integration in combination with global data fitting is used [32, 33].

In this paper, we compare evaluation tools using simulated kinetic data and determine how they can cope with different noise levels. Deviations from pseudo-first-order reaction kinetics can be ruled out when using simulated data allowing the comparison of different evaluation approaches without being flawed by device-specific error sources. In order to mimic real measurement data, noise was added to the simulated data. The effect of evaluating only a part of the association phase in contrast to evaluating the entire association phase is examined. In addition, these evaluation tools are used with experimental data of two antibodies measured with RIfS for comparison in order to apply the results of the evaluation of simulated data. For real data, it is important to check whether the goal of avoiding mass transport by reducing the immobilization level of the recognition element is achieved. This paper should be a guideline for BIA evaluation, point out what to look out for experimentally, and help with interpretation and verification of results.

## Materials and methods

### Materials

Common chemicals were purchased from Sigma-Aldrich (Taufkirchen, Germany) or Fluka (Neu-Ulm, Germany). The monoclonal IgG antibodies to amitriptyline (host mouse) clone 202 and clone TU-11 were purchased from Aviva Systems Biology Corporation (San Diego, USA) and antikoerper-online.de (Aachen, Germany) respectively. Poly(ethylene glycol) diamine (PEG-DA, MW 2000 Da) and ɑ-methoxy-ω-amino PEG (PEG-MA, MW 2000 Da) were purchased from Rapp Polymere (Tübingen, Germany). Phosphate-buffered saline (PBS) consisted of 150 mM sodium chloride and 10 mM potassium phosphate at pH 7.4. The solution used for regeneration of the sensor surface was guanidine hydrochloride (GdnHCl, 6 M, pH 1.5). RIfS glass transducers (1 cm × 1 cm) consisting of a 1-mm glass substrate with a layer of 10 nm Ta_2_O_5_ covered with 330 nm SiO_2_ on top were obtained from Schott AG (Mainz, Germany).

### Surface chemistry for RIfS transducers

The RIfS experiments were performed as described in [20]. The ligand nortriptyline (NRT) was immobilized on glass transducers using amine-coupling chemistry based on [34] and similar to [35]. The transducers (1 × 1 cm) were first cleaned for 30 s in KOH (6 M) solution, then washed with H_2_O. Next, they were cleaned and activated for 15 min using freshly prepared piranha solution (3:2 conc. H_2_SO_4_:H_2_O_2_ (30%)). After washing the transducers with H_2_O and drying under nitrogen, they were modified with 3-glycidyloxypropyl-trimethoxysilane (GOPTS) for 1 h. The transducers were cleaned with acetone and dried under nitrogen. The polymer mixture of PEG-DA and PEG-MA (1:1000) for the kinetic analysis was bound covalently onto the GOPTS layer using 20 μl PEG (4 mg/ml in dichloromethane DCM). After reacting overnight at 70 °C, the transducers were cleaned with H_2_O and dried under nitrogen. The amino functions of PEG-DA were transferred into carboxyl functions using 10 μl of dissolved glutaric acid (GA) (0.67 mg/μl GA in DMF). Each transducer was covered with another transducer in a DMF vapour-saturated chamber for at least 6 h. Afterwards, the transducers were cleaned with DMF and H_2_O and dried under nitrogen. Subsequently, NRT was immobilized on the sensor surface using N,N′-diisopropyl-carbodiimide (DIC) and N-hydroxysuccinimide (NHS) as coupling reagents. NHS (150 mg) and DIC (302 μl) were dissolved in 1 ml DMF, and the transducers covered with the solution in a DMF vapour-saturated chamber for 4 h. After cleaning with DMF and acetone, and drying under nitrogen, the transducers were incubated with NRT (2 mg/ml in H_2_O) in a water vapour-saturated chamber overnight. Then, the transducers were washed and dried under nitrogen.

### Measurement

RIfS was chosen for the BIA experiments because we have the best experience with this system and it allows the most experimental modifications. It is based on interference of white light at thin films [36]. At phase boundaries, part of the light is transmitted and part is refracted. The reflected partial beams superimpose resulting in an interference spectrum described in detail in [18, 36]. A change in optical thickness (nd; product of refractive index and physical thickness), which might be caused by antibody binding to antigen immobilized on the surface, results in a shift of the interference spectrum. Monitoring the optical thickness over time allows time-resolved detection [20] and typical binding curves are obtained.

To collect binding data, measurements are performed similar to [35, 37, 38]. First, the transducer surface was flushed with buffer (baseline). Then, different concentrations of the analyte (33 to 500 nM (antibody 150 kDa)) in PBS pH 7.4 were injected at a flow rate of 0.5 μl/s at room temperature without temperature control. The dimensions of the flow cell are 50 μm channel depth, 1 mm channel width, and 4 mm channel length. The complex was allowed to associate for 600 s and dissociate for 900 s. The sample and buffer were separated by air to prevent diffusion decreasing the sample concentration towards the end of the association and low sample concentration in the buffer during dissociation. The surfaces were regenerated with a 400 s injection of GdnHCl. Finally, another baseline was measured by flushing the cell with buffer again. Triplicate injections of each sample were flowed over the surface in random order.

### Simulation of kinetic data

Data were simulated using the 1:1 (Langmuir) binding model of BIAevaluation 4.1.1 with *k*_a_ = 1 · 10^4^ M^−1^s^−1^ and *k*_d_ = 1 · 10^−3^s^−1^. The maximum analyte binding capacity Rmax was set to 1 nm, and the bulk refractive index contribution RI was set to 0. The analyte concentrations for the simulations were 500 nM, 333 nM, 167 nM, 133 nM, 100 nM, 67 nM, and 33 nM. The association and dissociation times were set to 600 s and 900 s, respectively. A value was taken every 5 s which is the same rate as in the RIfS measurements. Noise was added by use of Matlab R2020b with an amplitude of 0.01 nm and 0.001 nm using random numbers with the following command where *y* is the simulated data and *r* the amplitude of noise:

$$ y\mathrm{Noisy}=y+2\cdotp r\cdotp \operatorname{rand}\left(\mathrm{length}(y),1\right)-2\cdotp r\cdotp 0.5 $$

The noise levels used were 0.001 nm and 0.01 nm, which is 1% of Rmax and about 1/14 of the smallest increase of optical thickness (0.14 nm for 33 nM). 0.01 nm noise is very large in comparison with real measurement data where the noise is typically around 0.001 nm noise.

Furthermore, the residuals obtained by mono-exponential fit of one of the experimental binding curves (67 nM clone 202, 25–525 s of the association phase) were added to the simulated curve of the same concentration.

### Data evaluation

Data evaluation was performed for the simulated and the experimental data. To evaluate the measured data, the first five data points and the last 20 data points of the association were left out (shown in Fig. S1). The methods used for the evaluation of the association phase were mono-exponential fit, derivative, and integration. The dissociation phase was evaluated with an exponential decay function and a linearization method. In addition, the BIAevaluation software was used to evaluate both phases. In order to compare how well different methods calculate the true rate constants for simulated data, relative deviations were calculated by

$$ \frac{k_{\mathrm{calculated}}-{k}_{\mathrm{true}}}{k_{\mathrm{true}}} $$

### Association

The association phase was evaluated using three different methods for three different areas of the association phase. For simulated data, first, the entire association phase was evaluated, then the first half, and finally, the association until an optical thickness of 0.5 nm was reached. For experimental data, the part of the association phase where the derivative showed linear behaviour was evaluated.

### Mono-exponential fit

The data of the association phase were fitted with the MnMolecular formula provided by Origin Pro 2021 (iteration algorithm: Levenberg Marquardt) which is *y* = *A*(1 − *e*^−*k*(*x* − *xc*)^). The theoretical integrated rate equation is

$$ \Gamma (t)={\Gamma}_{\mathrm{eq}}\left(1-{e}^{-{k}_{\mathrm{obs}}\cdotp t}\right) $$

where Γ(*t*) is the surface load capacity over time, Γ_eq_ the equilibrium surface load capacity, and *k*_obs_ the observed binding rate constant which also describes the curvature of the calculated fitting curve. In the case of RIfS, the measured signal is the difference in the optical thickness Δnd.

### *k*_obs_ linearization

The calculated *k*_obs_ for different concentrations are related to the rate constants by *k*_obs_ = *k*_a_ · *c* + *k*_d_ with the association rate constant *k*_a_ and the dissociation rate constant *k*_d_. By plotting *k*_obs_ vs. the antibody concentration, the association rate constant is obtained by linear regression performed by Origin (instrumental error weighting = 1/ei^2) where *k*_a_ represents the slope and *k*_d_ represents the *y*-axis intercept of the linear fit. For simulated data, error bars of *k*_obs_ represent the standard error of the fit, while for measured data, *k*_obs_ values are calculated as the mean of the triplicate measurements where the error bars indicate the standard deviation of the mean.

### Derivative

For the evaluation by derivative, the derivative of the surface load capacity dΓ(*t*)/d*t* (in this case d(Δnd)/d*t*) is plotted against the Γ(*t*) (here Δnd) where *k*_obs_ is obtained as the negative slope after linear regression.

$$ \frac{\mathrm{d}\Gamma (t)}{\mathrm{d}t}={k}_{\mathrm{a}}\cdotp c\cdotp {\Gamma}_{\mathrm{max}}-{k}_{\mathrm{obs}}\cdotp \Gamma (t) $$

### Integration

Integrating this rate equation gives

$$ \frac{\Gamma \left({t}_2\right)-\Gamma \left({t}_1\right)}{t_2-{t}_1}={k}_{\mathrm{a}}\cdotp c\cdotp {\Gamma}_{\mathrm{max}}-{k}_{\mathrm{obs}}\cdotp \frac{\int_{t_1}^{t_2}\Gamma (t)\mathrm{d}t\ }{t_2-{t}_1} $$

When starting from the beginning with *t*_1_ = 0 and Γ(0) = 0, this equation becomes

$$ \frac{\Gamma (t)}{t}={k}_{\mathrm{a}}\cdotp c\cdotp {\Gamma}_{\mathrm{max}}-{k}_{\mathrm{obs}}\cdotp \frac{\int_0^t\Gamma (t)\mathrm{d}t\ }{t} $$

**Fig. 1:**
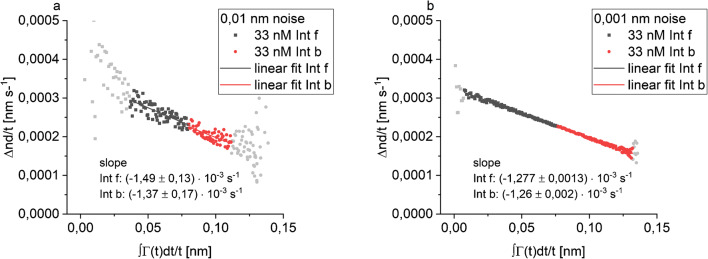
Evaluation by integration of the entire association phase of simulated data for 33 nM, with 0.01 nm noise integrated from the beginning (Int f, dark grey) and from the end (Int b, red) with values deviating from linearity masked (light grey) (**a**) and for simulated data for 33 nM with 0.001 nm noise (**b**)

The data of the association phase were integrated and divided by the integration time interval to obtain the *X* data. The integration of Δnd was performed with Origin (mathematical area). The difference between the optical thickness at the end of the integration and at the start of the integration divided by the time interval provided the *Y* data. A plot of Γ(*t*)/*t* vs. ${\int}_0^t\Gamma (t)\mathrm{d}t/t$ gives *k*_obs_ as the negative slope. The integration can be performed in two directions starting from the beginning (Int f) or from the end of the association phase (Int b). In both cases, a linear plot of the data is achieved with deviations from linearity for the first values (Fig. 1). For simulated data with 0.001 nm noise, the first ten values after integration were masked, for simulated data with 0.01 nm noise and experimental data, the values to be masked were determined by assessing which part of the data showed a linear behaviour. The integration was also preformed starting where half of the surface load capacity at the end of the association phase was reached.

### Dissociation

Data evaluation of the dissociation phase was performed with an exponential fit and a linearization method. The theoretical equation for dissociation is

$$ \Gamma (t)={\Gamma}_0{e}^{-{k}_{\mathrm{d}}\cdotp t} $$

where Γ_0_ is the surface load capacity at the beginning of the dissociation and *k*_d_ is the dissociation rate constant. The used function was ExpDecay1 in Origin $y={y}_0+{A}_1{e}^{-\frac{x-{x}_0}{t_1}}$ where *k*_d_ can be calculated from *t*_1_ by ${k}_{\mathrm{d}}=\frac{1}{t_1}$. *y*_0_ is the *y*-offset. Strict one-to-one interaction assumes that all bound analyte molecules can dissociate, but if the fitting function contains a *y*-offset, it allows analyte molecules to stay on the surface and thus describes back bonding.

For linearization, this function is transformed to

$$ \ln \left(\frac{\Gamma (0)}{\Gamma (t)}\right)={k}_{\mathrm{d}}\cdotp t $$

A plot of ln(Γ_0_/Γ(*t*)) vs. *t* gives *k*_d_ as the slope.

### BIAevaluation

For the evaluation with BIAevaluation, the beginning of the association phase was set to zero on the *x* and *y* scale (X-Transform, Y-Transform). The association phase was evaluated from 100 to 500 s for simulated data and until an optical thickness of 0.5 nm for clone 202 and 0.6 nm for clone TU-11 was reached. The dissociation phase was evaluated from 700 to 1400 s using Fit:Kinetics Simultaneous ka/kd and Fit:Kinetics Separate ka/kd with the 1:1 (Langmuir) model. For simultaneous ka/kd, BIAevaluation performs a global fit of association and dissociation of all concentrations. The chosen settings for the parameters were constant concentration; global fit of ka, kd, and Rmax; and local fit of RI. For Separate ka/kd, the dissociation phase is evaluated first. Settings were global fit of kd with local fit of R0 and offset, t0 was set constant to 605 s. Then, the obtained kd value is used as a constant for the global fit of ka of the association phase with constant concentrations and local fit of t0 and RI. For measured data, this setting for separate ka/kd resulted in too many fitted parameters; thus, t0 was fitted globally.

## Results and discussion

### Data evaluation of simulated data

**Fig. 2:**
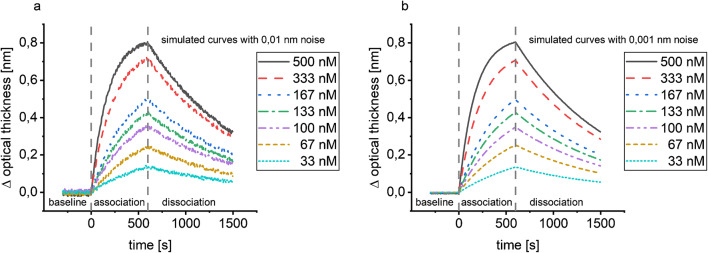
Simulated binding curves with 0.01 nm (**a**) and 0.001 nm noise (**b**). Simulations were performed using the 1:1 (Langmuir) binding model in BIAevaluation 4.1.1 for seven different analyte concentrations with 300 s baseline, 600 s association, and 900 s dissociation; random noise with different amplitudes was added using Matlab

The evaluation of simulated data allows the comparison of the evaluation methods, if a perfect one-to-one interaction can be safely assumed. The simulated binding curves are shown in Fig. 2. It is obvious that data simulated with 0.001 nm noise (Fig. 2b) represents data comparable to good kinetic measurements because the noise is less than 1/100 of the equilibrium surface load capacity of the smallest concentration, while data with 0.01 nm noise (Fig. 2a) would be considered in need of improvement of the experimental conditions.

### Rate constants

**Fig. 3:**
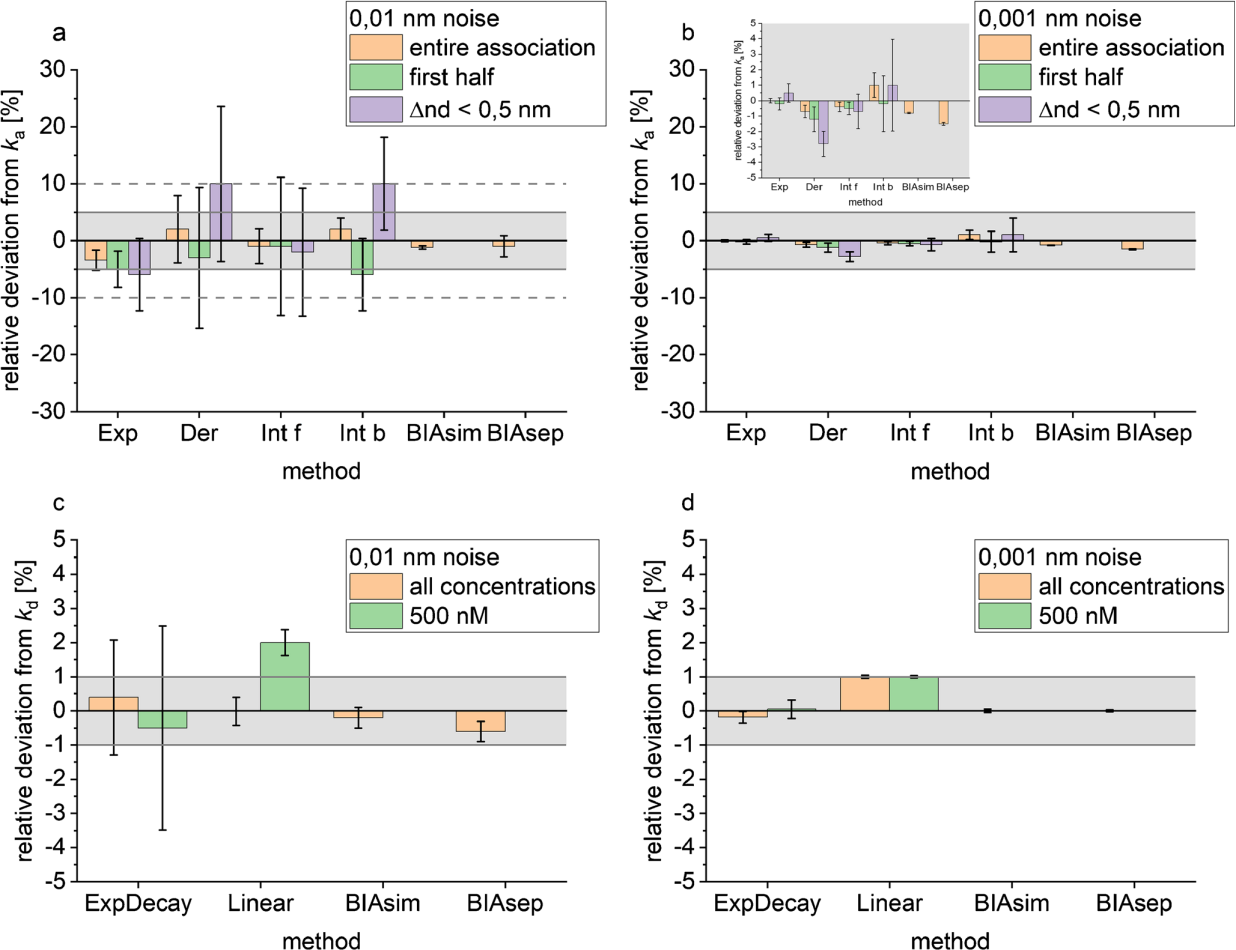
Relative deviations of the rate constant from the true value and their relative standard error of the fit for different evaluation methods. The relative deviations from the true association rate constant were calculated for simulated data with 0.01 nm noise (**a**), with 0.001 nm noise (**b**) and relative deviation of the dissociation rate constants from the true value for simulated data with 0.01 nm noise (**c**), and with 0.001 nm noise (**d**). The association rate constant was calculated by *k*_obs_ linearization after exponential fit (Exp) of the association phase, the derivative (Der), and forward and backward integration (Int f, Int b) for different evaluation areas (entire association phase (0–600 s), first half (0–300 s), and from the beginning of the association phase until a Δ optical thickness of 0.5 nm is reached (Δnd < 0.5 nm). Global fit was used for calculating *k*_a_ and *k*_a_ by BIAevaluation with simultaneous ka/kd (BIAsim) and separate ka/kd (BIAsep) for all concentrations. Dissociation rate constants were calculated by an exponential decay (ExpDecay) function and a linearization (Linear) with a global fit for all concentrations and for 500 nM

For the simulated curves with 0.01 nm noise, *k*_a_ values calculated by all methods are shown in Table 1. The calculated *k*_a_ values for the entire association deviate by less than 10% from the correct value for all methods as shown in Fig. 3a. In order to make a statement about the precision of the used methods, it is considered which ones hit the correct value up to a deviation of ± 5%. Both forward and backward integrations give *k*_a_ values deviating by less than 5% from the true value and the BIAevaluation methods as well. If only the first half of the association phase (300 s) is used for the calculation, all calculated *k*_a_ values deviate by less than 5% from the true value except for the backward integration. But if we take their standard errors of the fit into account, all used methods exceed a 5% deviation. (For BIAevaluation, the fitting region was not varied.) If the selection of the fitting area is based on the optical thickness and values are evaluated before reaching 0.5 nm, the calculated *k*_a_ values with their standard errors of the fit exceed a 10% deviation of the true *k*_a_ value.

**Table 1:** *k*_a_ in 10^4^ M^−1^ s^−1^ calculated with different methods for different fitting regions for simulated data with 0.01 nm noise. The *k*_a_ used for the simulation was 10^4^ M^−1^ s^−1^

Method	Entire association (0–600 s)	1st half of association (time)	Δnd < 0.5 nm	1st half of association (Δnd)	2nd half of association (Δnd)
Exp	0.966 ± 0.017	0.95 ± 0.03	0.94 ± 0.06	–	–
Der	1.02 ± 0.06	0.97 ± 0.12	1.10 ± 0.15	–	–
Int f	0.99 ± 0.03	0.99 ± 0.12	0.98 ± 0.11	1.1 ± 0.3	0.96 ± 0.15
Int b	1.02 ± 0.02	0.94 ± 0.09	1.10 ± 0.09	1.1 ± 0.2	1.05 ± 0.06
BIAsim	0.988 ± 0.003	–	–	–	–
BIAsep	0.990 ± 0.018	–	–	–	–

For the evaluation of the entire association phase, a *k*_a_ value deviating by less than 10% from the true value is obtained. As a deviation of 10% from the true value can be considered acceptable, it is concluded that all methods are in principle suitable for calculating *k*_a_, even if the signal is very noisy. The 5% deviation criterion shows that forward and backward integrations of the entire association phase seem to be superior to the evaluation by exponential fit and derivative in the case of noisy signals. The reduction of the fitting region leads to larger deviations from the true value and larger standard errors as the amount of data points is reduced.

For less noisy signals (0.001 nm), association rate constants are calculated correctly (less than 5% deviation) with all methods as shown in Fig. 3b. Table 2 shows that the *k*_a_ value closest to the *k*_a_ value simulated is clearly obtained, if the entire association phase is evaluated with the (mono-)exponential fit.

**Table 2:** *k*_a_ in 10^4^ M^−1^ s^−1^ calculated with different methods for different fitting regions for simulated data with 0.001 nm noise. The *k*_a_ used for the simulation was 10^4^ M^−1^ s^−1^

Method	Entire association (0–600 s)	1st half of association (time)	Δnd < 0.5 nm	1st half of association (Δnd)	2nd half of association (Δnd)
Exp	1.000 ± 0.0014	0.998 ± 0.004	1.005 ± 0.006	–	–
Der	0.993 ± 0.004	0.988 ± 0.008	0.972 ± 0.008	–	–
Int f	0.996 ± 0.003	0.995 ± 0.004	0.993 ± 0.011	0.99 ± 0.02	0.990 ± 0.017
Int b	1.01 ± 0.008	0.998 ± 0.018	1.01 ± 0.03	1.017 ± 0.015	1.016 ± 0.014
BIAsim	0.9920 ± 0.0004	–	–	–	–
BIAsep	0.9850 ± 0.0008	–	–	–	–

The dissociation rate constant is calculated correctly for all methods. They show less than 5% deviation from the true value for 0.01 nm noise (Fig. 3c), and less than 1% deviation for 0.001 nm noise (Fig. 3d). The closest values in the case of 0.01 nm noise are obtained with the linearization method performed for all concentrations and the BIAevaluation simultaneous ka/kd fit. With less noise (0.001 nm), the best results are obtained with the BIAevaluation separate ka/kd fit, BIAevaluation simultaneous ka/kd fit, and fitting an exponential decay function to the largest concentration 500 nM.

### Derivative

**Fig. 4:**
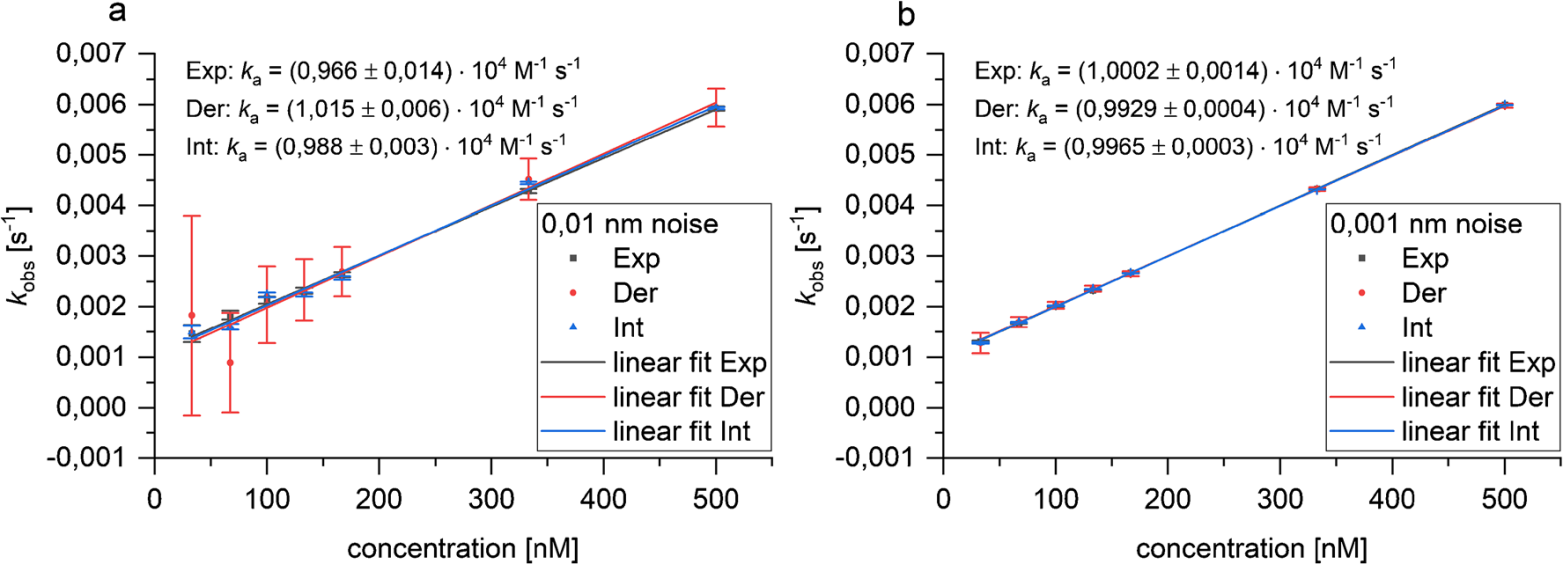
*k*_obs_ linearization for simulated curves obtained with different evaluation methods for the entire association phase (0–600 s) with **a** 0.01 nm noise and **b** 0.001 nm noise. *k*_obs_ were calculated by exponential fit (Exp), by derivative (Der), and by integration starting from the beginning of the association (Int). Error bars show the standard error of the fit. *k*_a_ is the slope

It is very interesting that the linearization methods show larger deviations especially when the part of the association evaluated is reduced. For the derivative, the noise largely affects the evaluation as the derivative increases the noise. If the derivative d(Δnd)/d*t* is plotted against the Δ optical thickness, this can result in problems with linear fit because the noise strongly affects the calculation of the observed rate constant, especially for small concentrations. Smaller concentrations are more affected by noise as the relative signal-to-noise ratio (*S*/*N*) is smaller. When the derivative for noisy signals is plotted against the signal (d(Δnd)/d*t* vs. Δnd plot) to obtain *k*_obs_ from the slope, a decreasing slope will become imperceptible due to the noise (Fig. S2a). If the *S*/*N* is very low, the slope is not significantly different from zero, showing that the evaluation by derivative is less suitable for noisy data. This is supported by the *k*_obs_ linearization. When comparing the *k*_obs_ linearization for the different methods (Fig. 4a), it becomes obvious that the *k*_obs_ calculated by derivative shows the largest standard error of the fit, especially for small concentrations. These problems do not occur if the *S*/*N* is large enough.

### Integration

The integration method can better cope with noisy signals as integration reduces noise. Figure 1 shows the integration of the entire association phase for the lowest concentration 33 nM for both noise levels. Unlike the derivative, no problem occurs with determining the slope for the data with 0.01 nm noise (Fig. 1a), if enough data points in the beginning of the integration are masked. The values calculated with forward and backward integration merge seamlessly as shown in Fig. 1b, which allows a nice control over a well-chosen evaluation area and cleanly determined slope.

However, especially for the first values of the integration, the noise still affects the calculated values even for the highest concentrations. The more the integration proceeds, the smoother the obtained curves. The deviation of the first values is less pronounced for the simulated data with less noise. But for strong noise, there is still a large deviation from the true values in the case of small concentrations. For the evaluation by integration, the *X* values are the integrated curve divided by the time interval of the integration. As the integration procedure reduces noise, the *X* values are not much affected by the noise in the signal. In contrast, the *Y* values represent the difference between the optical thickness at the end of the integration and the optical thickness at the start, resulting in the noise showing its effect in the *Y* values. When starting the integration from the end where this difference is lower because the curve flattens, the noise will affect the calculated values more, resulting in more values having to be masked to perform good linear fits. The effect of integration smoothing only shows after integration over a certain area. Therefore, the first values in the integration method should be masked for calculating *k*_obs_.

**Fig. 5:**
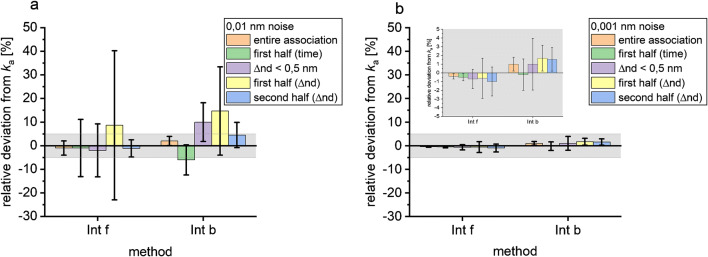
Relative deviations of *k*_a_ calculated by the integration method for different evaluation ranges from the true value for data with 0.01 nm noise (**a**) and with 0.001 nm noise (**b**). Error bars indicate the relative error of the fit

The integration method offers a variety of possible starting points. Integration can be started at the beginning of the association phase, but also at the end or in the middle. Figure 5 shows that using all data points (evaluating the entire association phase) and starting in the beginning of the association phase give the best results for both noise levels. Starting the evaluation in the middle where half of the final Δnd is reached towards the end (Int f second half) gets a *k*_a_ value very close to the true value for very noisy signals (Fig. 5a). Starting in the middle and integrating towards the beginning of the association phase come very close for less noisy signals (Int b first half in Fig. 5b).

Having the previous argumentation in mind that noise has less effect on the start of the integration where the signal increase is large, it makes sense to start the integration where the signal increase is larger. Thus, the integration should be started at the beginning or in the middle where half of the final Δ optical thickness is reached, but not at the end of the association phase. For real experiments, choosing the middle as the starting point would have the advantage that unwanted effects of the fluidics that might occur in the beginning of the association can be omitted.

### Comparison of methods

The evaluation of simulated binding curves shows that all considered methods are capable of calculating binding constants as they provide rate constants close enough to the true value. Table 3 shows a rating of the methods related to different aspects. All methods provide very good results, if the noise is small compared to the signal, which should be the case, if the experimental design is good. Only if the noise is very large compared to the signal do some methods lack accuracy. The comparison of very noisy and less noisy signal showed that the derivative leads to large errors, if the signal is very noisy, whereas the integration can better cope with noise. The integration can deal best with noisy data, because the integration method shows a smoothing effect. The BIAevaluation software also gives values very close to the true *k*_a_ value for noisy data.

**Table 3:** Rating of evaluation methods for the calculation of the association rate constant

Method	Exp	Der	Int f	Int b	BIAevaluation
Robustness against noise	−	−	+ +	+	+ +
Robustness against choice of evaluated region	+	−	+	−	Not examined
Independence of masking of data	+	+	−	−	+

The evaluation of different parts of the association phase shows that the best results are achieved when using the entire association phase. Using only parts of the association phase leads to a loss of information and thus to less precise results. In contrast, when evaluating experimental data, it can be advantageous to select the part of the association phase that matches the model. Exponential fit and forward integration can better deal with the reduction of the fitting area than derivative and backward integration. In general, the choice of the evaluated region is very important and as many data points as possible should be included.

In the case of the integration method, it was necessary to mask the first data points in the plot of Γ(*t*)/*t* vs. ∫Γ(*t*)d*t*/*t* in order to obtain good results whereas the other methods did not require additional data selection after the region for evaluation was chosen.

When residuals of the curve fitting of an experimental curve were added to a simulated binding curve, *k*_obs_ was successfully determined by mono-exponential fit, derivative, and forward and backward integration (Fig. S3). The obtained *k*_obs_ values were sufficiently close to the simulated value deviating by less than 10% (Table S1). Thus, it is concluded that these evaluation methods should also be suitable for data with experimental noise. As the experimental data shown here can be compared to the simulated data with simulated noise of an amplitude of 0.001 nm with respect to their noise levels, similar results are expected when evaluating binding curves to which the residuals of experimental data are added.

### Data evaluation of measured data

**Fig. 6:**
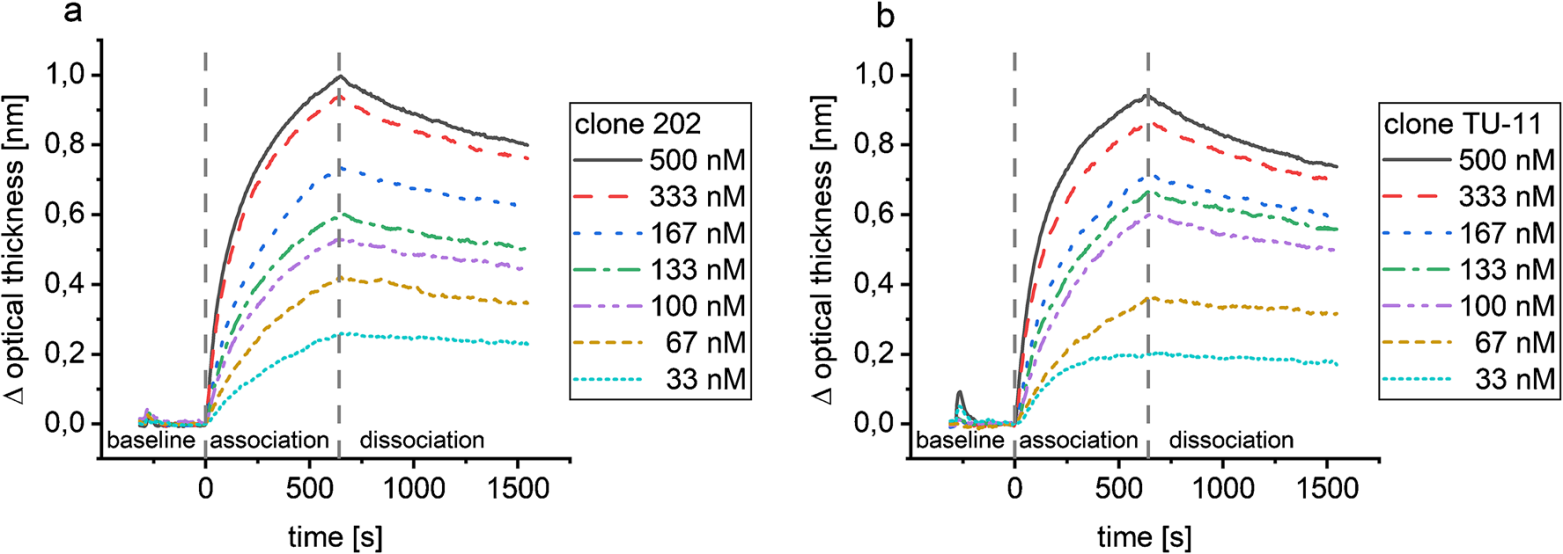
Example RIfS measurements for each concentration of antibody clone 202 (**a**) and clone TU-11 (**b**). The baseline with PBS was followed by the injection of multiple analyte anti-amitriptyline antibody concentrations (association) and the dissociation of the antibody in PBS

Example binding curves of the RIfS measurements are shown in Fig. 6. To check if one-to-one interaction is applicable, the natural logarithm of the derivative of the optical thickness of the association phase ln(d(Δnd)/d*t*) can be plotted against time, which should be linear [32]. In Fig. S4, these checks are shown for an example 500 nM measurement. The plot is linear for the association from 20 to 110 s for clone 202 and to 170 s for clone TU-11, respectively. For the dissociation phase, ln(Γ(0)/Γ(*t*)) vs. time is plotted which deviates for both antibodies from a linear curve.

As the check for one-to-one interaction shows, one-to-one interaction cannot be assumed for the entire association phase. The derivative plotted vs. time is linear for a part of the association in the beginning, indicating that in this part using the model for pseudo-first-order reaction kinetics is possible. This simple model allows the comparison of the antibodies.

In the dissociation phase, a deviation from linearity is observed as shown in Fig. S4c and d, too. This shows that the criteria for a pseudo-first-order model are not completely met. Nevertheless, the deviation is rather small; the data only show a slight curvature.

This deviation from pseudo-first-order kinetics in the dissociation might be caused by the avidity of the antibody. If the antibody is bound to the surface with both paratopes, the dissociation will not follow pseudo-first-order kinetics and it might be possible that the antibody does not completely dissociate.

Even though the immobilization level was reduced to achieve low amounts of antigen on the surface and the flow rate was fast (30 μl/min), one-to-one interaction is not applicable. This result shows how difficult it is to design the experiment in the right way. A reason for the deviation could be the bivalency of the antibody or inhomogeneities. In this case, it was impossible to immobilize the antibody instead of the antigen because the binding of the antigen is too small to detect. Thus, avidity effects cannot be ruled out. Nevertheless, high-quality binding curves were obtained and evaluated.

The plot of the derivative vs optical thickness shows that in the beginning of the association, the derivative can be approximated by a linear fit (Fig. S5a and c). For larger concentrations, it seems as if there is a kink in the derivative when an optical thickness of 0.5 nm for clone 202 and 0.6 nm for clone TU11 is reached. This linear part of the derivative was used for a linear fit to obtain *k*_obs_. In the *k*_obs_ vs. *c* plot shown in Fig. S5b and d, the *k*_obs_ values for all concentrations lie on a straight line. Only the smallest concentration shows a larger deviation than the others for clone 202.

Evaluating the part of the association where the derivative shows linear behaviour with the model for pseudo-first-order kinetics is reasonable as for this part a one-to-one interaction can be assumed. The linearity of the *k*_obs_ vs. *c* plot confirms that this model can be used.

If only the part of the association phase where the derivative is linear is evaluated (Δnd < 0.5/0.6 nm), the obtained values for *k*_a_ are significantly larger than if the entire association phase or the first half (time) is evaluated. When looking at the derivative of the large concentrations, it becomes obvious that evaluating more than the part up to the kink will lead to lower *k*_obs_ values for these concentrations, while the *k*_obs_ values for the small concentrations will remain the same as these concentrations do not reach an optical thickness of 0.5 or 0.6 nm. As only the larger *k*_obs_ values will decrease, this results in smaller slope when plotting *k*_obs_ vs. *c*, and consequently to a smaller *k*_a_ value. Fitting the entire association phase gives the average rate constant.

The kink in the derivative might be caused by an inhomogeneous surface, with some easily accessible surface sites to which the antibody can bind faster and some surface sites that are harder to reach and to which the antibody only binds when the easily accessible sites are occupied. This would mean that the antibody binds to different surface sites with different rate constants. Until a certain optical thickness is reached, the faster rate constant dominates.

**Fig. 7:**
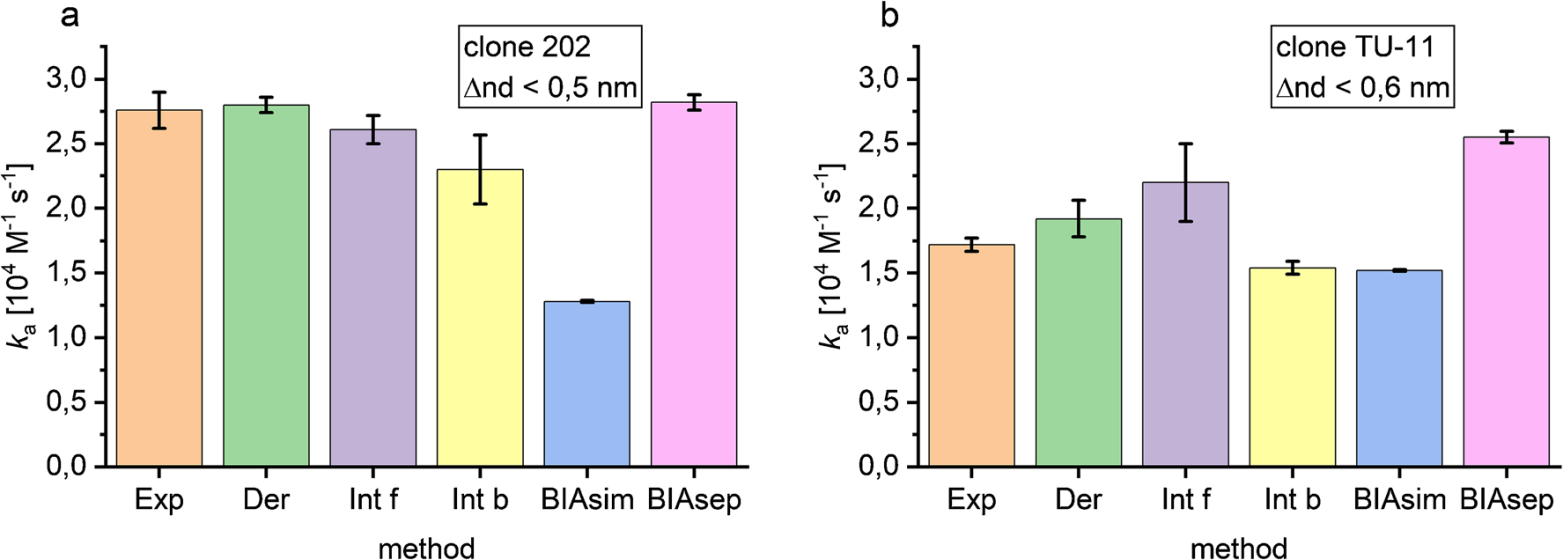
Association rate constants calculated with different methods for antibody clone 202 (**a**) and clone TU-11 (**b**). The evaluated area was based on the linear behaviour in the derivative, i.e. until a Δ optical thickness of 0.5 nm was reached for clone 202 and 0.6 nm for clone TU-11. Methods used were mono-exponential fit (Exp), derivative (Der), forward and backward integration (Int f, Int b), simultaneous ka/kd fit in BIAevaluation 4.1.1 (BIAsim), and separate ka/kd fit in BIAevaluation (BIAsep). Error bars show the standard error of the fit

The association rate constant was calculated with different methods for the part of the association phase showing linear behaviour in the derivative, and the obtained results for the *k*_a_ values are shown in Fig. 7. For both antibodies, the methods exponential fit, derivative, and forward integration provided the same *k*_a_ values, for clone 202 on average 2.4 ∙ 10^4^ M^−1^ s^−1^ and for clone TU-11 on average 1.9 ∙ 10^4^ M^−1^ s^−1^. The backward integration and BIAevaluation simultaneous ka/kd fit gave smaller *k*_a_ values, whereas the BIAevaluation separate ka/kd fit gave larger *k*_a_ values. In both cases, the maximum *k*_a_ value, which was calculated with 2.8 ∙ 10^4^ M^−1^ s^−1^ for clone 202 and 2.6 ∙ 10^4^ M^−1^ s^−1^ for clone TU-11, were obtained by the BIAevaluation separate ka/kd fit. All evaluation methods show that clone 202 has a larger association rate constant than clone TU-11, except the evaluation method BIAevaluation simultaneous ka/kd fit. The minimum *k*_a_ value was for both antibodies calculated with the BIAevaluation simultaneous ka/kd fit with 1.3 ∙ 10^4^ M^−1^ s^−1^ for clone 202 and 1.5 ∙ 10^4^ M^−1^ s^−1^ for clone TU-11.

As the evaluation methods (mono-)exponential fit, derivative, and integration all give similar rate constants, it can be concluded that the assumption of pseudo-first-order kinetics was justified. It is quite surprising that the two evaluation methods provided by the BIAevaluation software gave different results. The BIAevaluation software with its global fitting routines does not provide consistent data in this case. As all methods except BIAevaluation simultaneous ka/kd fit show a larger *k*_a_ value for antibody clone 202, it is concluded that this antibody associates faster.

**Fig. 8:**
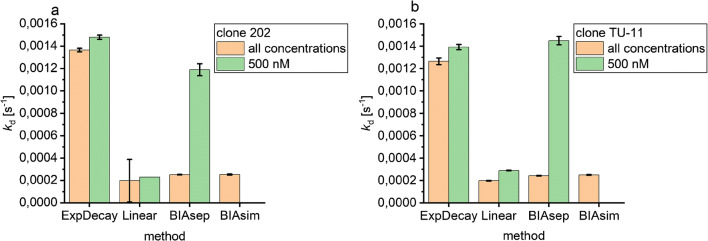
Dissociation rate constants *k*_d_ calculated with different methods for antibody clone 202 (**a**) and antibody clone TU-11 (**b**). *k*_d_ was calculated with an exponential decay function (ExpDecay), a linearization method (Linear), the separate ka/kd fit in BIAevaluation 4.1.1, and the simultaneous ka/kd fit in BIAevaluation for all concentrations and the highest concentration 500 nM. Error bars indicate the standard deviation of the fit

To obtain the dissociation rate constants, all concentrations and the highest concentration were evaluated with four methods. The dissociation rate constants calculated by exponential fit and BIAevaluation with separate ka/kd fit for 500 nM are five to seven times larger than those calculated by other methods (Fig. 8). The average *k*_d_ value obtained with the exponential fit and the BIAevaluation separate ka/kd fit for 500 nM is 1.3 ∙ 10^−3^ s^−1^ for clone 202 and 1.4 ∙ 10^−3^ s^−1^ for clone TU-11, while the average *k*_d_ value obtained with the other methods is 2.3 ∙ 10^−4^ s^−1^ for clone 202 and 2.5 ∙ 10^−4^ s^−1^ for clone TU-11.

The difference between using the exponential decay function and the other methods is that the *y*-offset in the fitting function allows back bonding. This means that not all antibodies need to dissociate from the surface. If only the 500 nM concentration is used for the BIAevaluation separate ka/kd fit, the fit performed is basically the same as with the exponential decay function in Origin. BIAevaluation also has an offset implemented in its function which then allows the antibody not to fully dissociate. As the number of fitted parameters cannot exceed 31 in BIAevaluation, this offset was set to a global fit instead of a local fit as in Origin. If this global fit is performed for all concentrations, this fit effectively leads to the offset being very small as the best value for all concentrations has to be found. If it is locally fitted on the other hand, larger offsets are obtained for the higher concentrations. The smaller dissociation rate constants obtained with the other methods show the result that is obtained if the antibody has to dissociate completely. All in all, the *k*_d_ values obtained for the two antibodies show that they do not significantly differ in dissociation.

The calculation of rate constants for experimental data shows that different rate constants are obtained for different methods, but they are in a similar order of magnitude. If no strict one-to-one interaction can be assumed, but the data is still evaluated with this model, an average rate constant is obtained. The rate constant that more adequately describes the pseudo-first-order association rate constant is calculated by selecting only the linear part of the derivative as the fitting region. A linear slope for a part of the association phase indicates that during this time the interaction follows the one-to-one model which allows the calculation of *k*_a_ for this part.

## Conclusion

In summary, we show that different methods can be used for the evaluation of binding curves. The evaluation of simulated binding curves shows that different methods can be used to calculate the correct binding rate constants. The obtained *k*_a_ values when using linearization methods (derivation and integration) show larger deviations from the true values than other methods. Calculations by derivative and backward integration are less reliable since they are affected by noise. The values closest to the true *k*_a_ value are obtained using BIAevaluation or by forward integration of the entire association phase for very noisy data. For simulated data with noise in the range of real measurements, the values closest to the true *k*_a_ value are obtained using the fit with an exponential function or by evaluating with the forward integration followed by a *k*_obs_ linearization. Thus, the integration is strongly recommended.

For experimental data, association and dissociation rate constants can successfully be determined although no strict pseudo-first-order kinetic could be assured. Adapting the fitting region shows a change in the association rate constant. On the basis of the derivative, the part of the association where pseudo-first-order kinetics is followed is selected and evaluated. The rate constants calculated with different methods are of the same order of magnitude. Finally, the results here suggest that looking at the derivative is very important as different rate constants become visible and it allows the selection of the part that follows pseudo-first-order kinetics. Evaluation with different methods of the same part of the association phase gives the same *k*_a_ value for exponential fit, derivative, and integration.

The results show that it is important not to rely on black box software, but instead to critically assess the data. We hope that this work will aid other researchers to generate more reliable data from BIA without having to rely on proprietary solutions.

## Supplementary information

ESM 1 (DOCX 796 kb)
