# Supplementary material for: Comparison of methods for quantitative biomolecular interaction analysis
Source: Anal Bioanal Chem. 2021 Sep 10;414(1):661–73. doi: 10.1007/s00216-021-03623-x (PMC8748344; doi:10.1007/s00216-021-03623-x)
Supplement: Supplementary file 1 — (DOCX 796 kb) [file 216_2021_3623_MOESM1_ESM.docx]

Supplementary Information (SI)

Comparison of methods for quantitative biomolecular interaction analysis

Analytical and Bioanalytical Chemistry

Monika Conrad, Peter Fechner, Günther Proll, Günter Gauglitz

monika.conrad@uni-tuebingen.de

Institute of Physical and Theoretical Chemistry (IPTC), Eberhard Karls Universität Tübingen, Auf der Morgenstelle 18, 72076 Tübingen, Germany


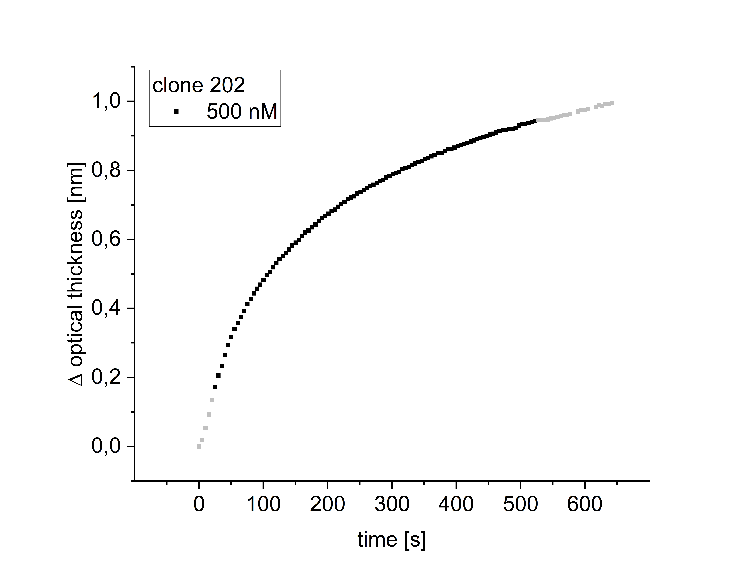


**Fig. S1** Example for evaluated region of measured binding curves. Association phase of 500 nM clone 202. First five and last 20 data points were left out (grey).


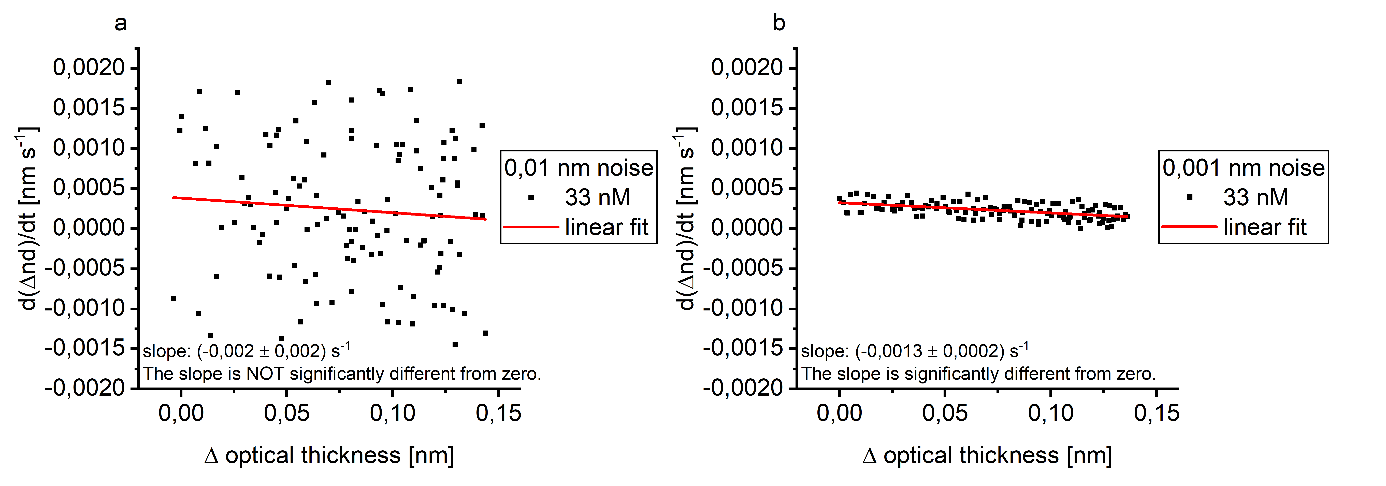


**Fig. S2** Plot of derivatives $d(\Delta nd)/dt$ vs. Δ optical thickness of simulated association phase (0 – 600 s) for 33 nM with 0.01 nm noise (a) and 0.001 nm noise (b). The slope (red) is the negative k_obs_ value obtained by linear regression.


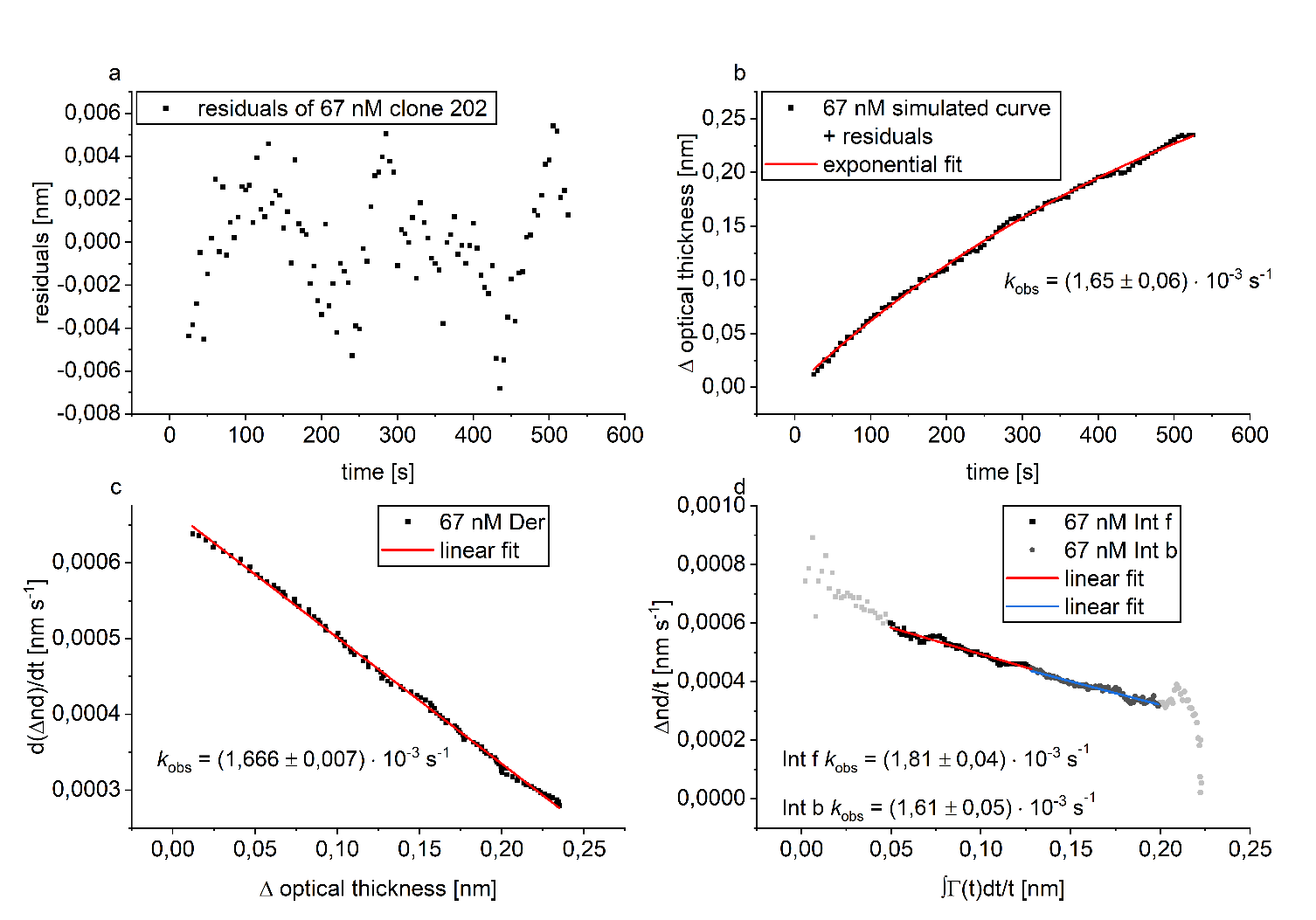


**Fig. S3** Evaluation of simulated curve with residuals added as noise. Residuals of mono-exponential curve fit of 67 nM clone 202 (a) was added to simulated data for 67 nM (*k*_obs_ = 0.00167 s^-1^) and evaluated by mono-exponential (b), derivative (c), and integration (d). Grey points were masked for the curve fitting.

**Table S1** Relative deviations of the calculated *k*_obs_ from the true value for simulated curves with added residuals

| Method | Relative deviation from *k*_obs_ [%] |
| --- | --- |
| Exp | 1 ± 3 |
| Der | -0.2 ± 0.4 |
| Int f | 8 ± 3 |
| Int b | -4 ± 3 |


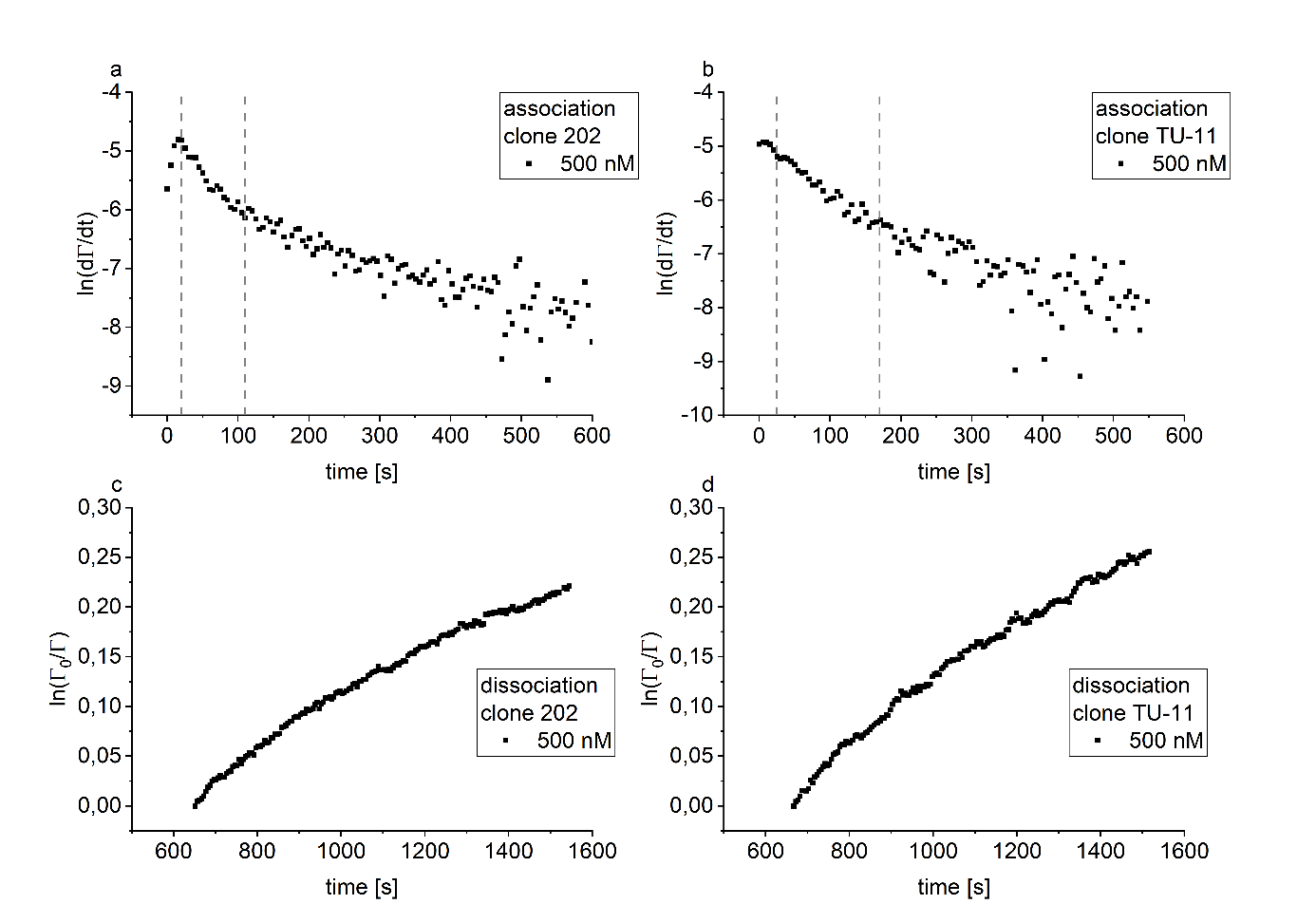


**Fig. S4** Checks for one-to-one interaction for association and dissociation for two antibodies; a) association of 500 nM clone 202, b) association of 500 nM of clone TU-11, c) dissociation of 500 nM of clone 202, d) dissociation of 500 nM of clone TU-11


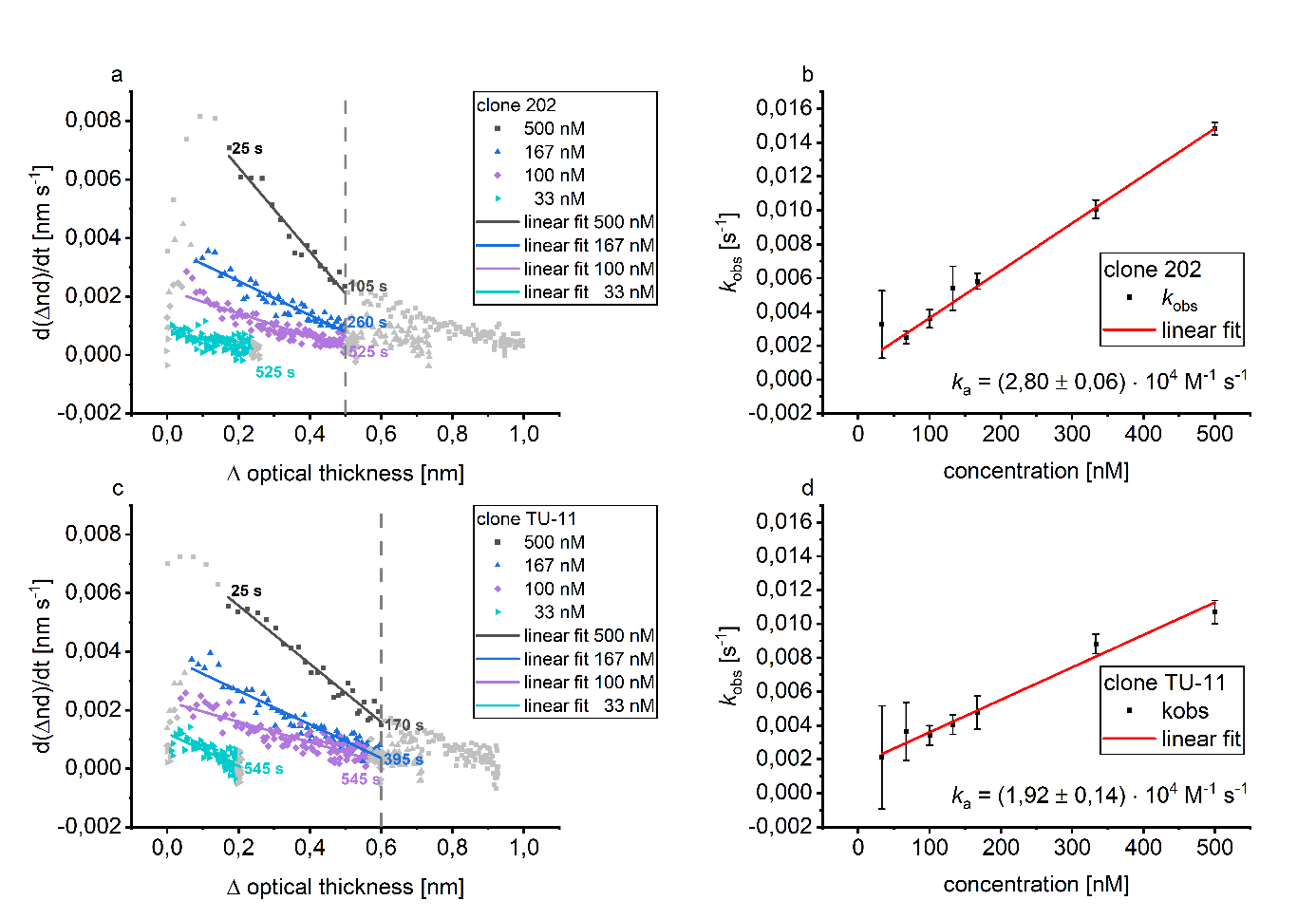


**Fig. S5** Example derivatives of the association phase of measurements of clone 202 (a), linear fit is started after 25 s of the association phase and was performed until an optical thickness of 0.5 nm is reached, last 20 values of the association phase were masked. k_obs_ linearization for clone 202 (b), k_obs_ calculated from the slope of the derivative as the mean of triplicate measurements. Error bars are standard deviations of the mean. The mean value with standard deviation of triplicates of k_obs_ was plotted vs the concentration and the association rate constant k_a_ obtained as the slope of linear regression. Example derivatives for clone TU-11 (c), linear fit until an optical thickness of 0.6 nm was reached. k_obs_ linearization for clone TU-11 (d)
